# Residential Racial Isolation and Spatial Patterning of Hypertension in Durham, North Carolina

**DOI:** 10.5888/pcd16.180445

**Published:** 2019-03-28

**Authors:** Mercedes A. Bravo, Bryan C. Batch, Marie Lynn Miranda

**Affiliations:** 1 Department of Statistics, Rice University, Houston, Texas; 2 Children’s Environmental Health Initiative, Rice University, Houston, Texas; 3 Department of Medicine, Endocrinology, Metabolism, and Nutrition, Duke University School of Medicine, Durham, North Carolina

## Abstract

**Introduction:**

Neighborhood characteristics such as racial segregation may be associated with hypertension, but studies have not examined these relationships using spatial models appropriate for geographically patterned health outcomes. The objectives of our study were to 1) evaluate the geographic heterogeneity of hypertension; 2) describe whether and how patient-level risk factors and racial isolation relate to geographic heterogeneity in hypertension; and 3) examine cross-sectional associations of hypertension with racial isolation.

**Methods:**

We obtained electronic health records from the Duke Medicine Enterprise Data Warehouse for 2007–2011. We linked patient data with data on racial isolation determined by census block of residence. We constructed a local spatial index of racial isolation for non-Hispanic black patients; the index is scaled from 0 to 1, with 1 indicating complete isolation. We used aspatial and spatial Bayesian models to assess spatial variation in hypertension and estimate associations with racial isolation.

**Results:**

Racial isolation ranged from 0 (no isolation) to 1 (completely isolated). A 0.20-unit increase in racial isolation was associated with 1.06 (95% credible interval, 1.03–1.10) and 1.11 (95% credible interval, 1.07–1.16) increased odds of hypertension among non-Hispanic black and non-Hispanic white patients, respectively. Across Durham, census block-level odds of hypertension ranged from 0.62 to 1.88 among non-Hispanic black patients and from 0.32 to 2.41 among non-Hispanic white patients. Compared with spatial models that included patient age and sex, residual heterogeneity in spatial models that included age, sex, and block-level racial isolation was 33% lower for non-Hispanic black patients and 20% lower for non-Hispanic white patients.

**Conclusion:**

Racial isolation of non-Hispanic black patients was associated with increased odds of hypertension among both non-Hispanic black and non-Hispanic white patients. Further research is needed to identify latent spatially patterned factors contributing to hypertension.

**Summary**

**What is already known on this topic?**

Neighborhood characteristics are possible underlying causes of health and health disparities among racial/ethnic groups in the United States.

**What is added by this report?**

Few studies have examined relationships between local measures of neighborhood racial isolation and hypertension, a prevalent chronic disease. We identified US Census blocks with the highest overall odds of hypertension in Durham, North Carolina, and estimated cross-sectional associations between racial isolation and odds of hypertension.

**What are the implications for public health practice?**

Information can be used to inform targeted interventions to address risk factors for developing hypertension or manage existing hypertension.

## Introduction

Hypertension, a chronic health condition affecting approximately 1 in 3 US adults, increases the risk of myocardial infarction, stroke, heart failure, kidney disease, vision loss, and peripheral artery disease (1,2). In the United States, hypertension is most prevalent among black people (3), a disparity that persists even after adjustment for individual-level risk factors (4). Increasingly, neighborhood characteristics are implicated as possible underlying causes of health disparities observed across racial/ethnic groups. In the United States, place of residence is strongly patterned by race/ethnicity, and a growing body of evidence links neighborhood environmental characteristics with a range of health outcomes. Nonetheless, only a few studies have examined relationships between neighborhood characteristics and hypertension.

Racial residential segregation is posited to be a fundamental cause of health disparities. Racial residential segregation of black people refers to the degree to which black people live separately from other racial/ethnic groups (5). Through the concentration of poverty and poor physical and social environments, racial residential segregation results in distinctive ecologic environments for black people that may underlie racial health disparities (6). Racial residential segregation is linked with various adverse health outcomes, including type 2 diabetes (7), preterm birth (8), infant mortality (9), and all-cause mortality (10).

Two studies of metropolitan-level segregation and hypertension found that adults residing in more segregated areas were more likely to be hypertensive than those living in less segregated areas (11,12); in one study this association was observed among black people but not white people (12). A study in New York City that used a local measure (as opposed to a city or metropolitan measure) of segregation found that non–US-born black people aged 65 or older residing in highly segregated neighborhoods were less likely to be hypertensive than their counterparts in neighborhoods with low levels of segregation, but this association was not observed among US-born black people aged 65 or older (13). In another study, black–white disparities in the prevalence of hypertension were attenuated in a racially integrated, low-income Baltimore neighborhood, suggesting that exposures associated with neighborhood environment explained some of the racial differences in hypertension observed in nationally representative samples (14). More recently, in 2017, a longitudinal study with follow-up over 25 years found that, among black people, moving from a less segregated to more segregated neighborhood was associated with a rise in systolic blood pressure (15).

Previous work examining cross-sectional associations of local measures of racial residential segregation with hypertension used aspatial statistical models that assumed independence among geographic units used to define a person’s living space. Ignoring spatial dependency in a health outcome may lead to underestimation of standard errors, producing narrow confidence intervals and, potentially, incorrect inference (16).

The objectives of our study were to use aspatial and spatial regression techniques to 1) evaluate the geographic heterogeneity of hypertension; 2) describe whether and how patient-level risk factors and racial isolation relate to geographic heterogeneity in hypertension; and 3) examine cross-sectional associations of hypertension with racial isolation.

## Methods

We used electronic health records from the Duke University Health System in Durham, North Carolina. We use a local spatial measure of racial isolation that represents 1 dimension of racial residential segregation and helps to overcome the shortfalls of simple measures of racial composition (eg, percentage of black residents) (17). We focused on the racial isolation of non-Hispanic black people because, compared with other dimensions of racial residential segregation (eg, evenness, the differential distribution of a population across a geographic unit), racial isolation may be more closely linked to health by serving as a proxy for the concentration of multiple disadvantages into a single ecological space (18).

The study area consisted of 5,029 census blocks composing Durham County, North Carolina. The Durham County population is 37.5% non-Hispanic black, 42.1% non-Hispanic white, and 13.5% Hispanic (19).

### Patient data

We obtained electronic health records from the Duke Medicine Enterprise Data Warehouse for 377,556 unique persons who were patients of a Duke Medicine provider at any time from January 1, 2007, through December 31, 2011. Using ArcGIS software (Esri), we street-geocoded the residential address of each patient to link patients to a 2010 census block. Of 361,434 patients with valid addresses, 88% were geocoded. We restricted the geocoded data set to patients residing in Durham County (remaining n = 243,837) and removed data on patients whose records consisted only of laboratory test results (remaining n = 243,820). We excluded patients younger than 18 years or with missing information on age, race/ethnicity, or sex (remaining n = 171,520). We further restricted our analysis to patients who were either non-Hispanic black or non-Hispanic white (remaining n = 147,359) and resided in census blocks with a nonzero population (remaining n = 147,351).

Patients were defined as having hypertension on the basis of the following *International Classification of Diseases, Ninth Revision, Clinical Modification* (ICD-9) codes: 401.0, 401.9, 402.00, 402.01, 402.10, 402.11, 402.91, 403.00, 403.01, 403.10, 403.11, 404.00–404.03, 404.10–404.13, 404.90–404.93, 405.01, 405.09, 405.11, 405.19, 405.91, 405.99, and 437.2. We constructed a hypertensive status indicator equal to 1 if a patient ever received a positive diagnosis during the study period and 0 otherwise. We constructed maps to show, by quintile, the proportion of patients with a hypertension diagnosis during the study period in each census block. 

This research was approved by the institutional review boards at Duke University and Rice University.

### Racial isolation

Using 2010 census data, we calculated block-level racial isolation scores by accounting for the population composition in the index block along with adjacent blocks. We thus included neighboring blocks in surrounding counties in our adjacency structure.

The local spatial measure of racial isolation, described in detail elsewhere (8), ranges from 0 to 1: 0 indicates that the neighborhood environment is 100% non-black (no isolation), and 1 indicates that it is 100% black (complete isolation). We linked information on block-level racial isolation with patient data based on each patient’s block of residence.

### Statistical analysis

We computed descriptive statistics for the study sample. To inform the use of race-stratified models, we evaluated whether the racial isolation exposure distributions of non-Hispanic black patients and non-Hispanic white patients overlapped. To some degree, both populations had nonoverlapping neighborhoods (Appendix); that is, non-Hispanic black and non-Hispanic white patients tended to reside in different blocks and have different residential environments. Consequently, we chose to proceed with a race-stratified modeling approach.

#### Geographic heterogeneity of hypertension

We evaluated the geographic heterogeneity of a patient-level hypertension diagnosis by comparing 4 patient-level logistic regression models with the following: 1) no random effects (standard model); 2) unstructured block-level random effects only (ν*_j_* in Equation 1, the random-intercept model); 3) spatially structured block-level random effects only (υ*_j_* in Equation 1, the spatially structured model); and 4) both structured and unstructured block-level random effects (υ*_j_* + ν*_j_* in Equation 1, the convolution model). Thus, the convolution model was of the following form:

$log\left(\frac{{\hat{p}}_{ij}}{1-{\hat{p}}_{ij}}\right)$= ${\hat{\beta }}_{0}+{\hat{\beta }}_{1}x_{ij}+{\hat{\beta }}_{2}z_{j}+\nu_{j}+\upsilon_{j}$ (Equation 1)

where *p̂_ij_* is the fitted probability of patient *i *in block *j *having hypertension, *x_ij_* is a vector of individual-level covariates (eg, age, sex) for patient *i* in block *j*; *z_j_* is a block-level covariate for block *j* (eg, racial isolation); and ν*_j_* and υ*_j_* are the unstructured and spatially structured block-specific random effects for block *j*, respectively.

Models with random effects are based on the hypothesis that patients in the same block share sources of unobserved variation in hypertension. The unstructured random effect assumes that blocks are independent across geographic space, whereas the spatially structured random effect assumes that hypertension in blocks nearer to each other is more similar. This term reflects sources of unobserved heterogeneity that vary locally (“clustering”). The unstructured random effects (ν*_j_*) are assigned a normal prior with unknown variance.

For the spatially structured block-level random effects (υ*_j_*), we assumed a Besag–York–Mollie specification (20), modeled by using an intrinsic conditional autoregressive (iCAR) structure:

υj|υk≠j~Normal(mj,ϑυ2#N(j)) (Equation 2)

$m_{j}=\frac{\Sigma_{k\in N(j)}\upsilon_{j}}{\#N(j)}$

where *m_j_* is the mean of the spatial random effects of blocks neighboring block *j*, and *#N(j)* is the number of blocks neighboring block *j* (21).

The variances of the unstructured and spatially structured random effects represent unknown hyperparameters, with priors for the precision taken from γ distributions with shape and scale equal to 1 and 0.0005, respectively. For all models, we assigned vague normal (0, 1000) priors to the parameters for patient risk factors and racial isolation.

We fit 3 model specifications, including a null model, a model adjusting for patient-level risk factors for hypertension (age, sex), and a model adjusting for patient-level risk factors and racial isolation. We used these model specifications to examine how residual geographic heterogeneity (ie, the variance of the block-level spatially structured random effects) in hypertension changes after the addition of patient-level risk factors and racial isolation. We conducted model selection by using the deviance information criterion (DIC) (22), with differences in DIC of 5 or less considered not meaningful.

We calculated the percentage change in residual geographic heterogeneity by sequentially comparing the null, patient-level risk factor, and patient-level risk factor plus racial isolation models.

#### Cross-sectional association of hypertension and racial isolation

We used the racial isolation index of non-Hispanic black patients in both the white and black race-stratified models and tabulated cross-sectional associations per 0.20-unit increase in racial isolation. We selected the regression model that included patient-level risk factors and racial isolation based on the DIC. We then applied a map decomposition technique (23) to explore the relative contribution of racial isolation versus the unstructured and spatially structured random effects to odds of hypertension at the block level. For example, for a given block, the component odds for racial isolation is equal to the exponentiated fixed-effect estimate multiplied by the standardized racial isolation value for that block. This quantity represents the contribution of racial isolation to odds of hypertension for the average patient in the index block. Mapping the component odds enables visualization of the geographic distribution of odds of hypertension and the extent to which local odds may be driven by racial isolation versus unobserved sources reflected in the random effects.

#### Sensitivity analysis

We compared the Watanabe–Akaike information criterion (24) with the DIC to select our model. In place of the local spatial measure of racial isolation, we examined cross-sectional associations between hypertension and the block-level proportion of non-Hispanic black residents. Cross-sectional associations estimated between racial isolation and hypertension may be subject to confounding from factors for which we did not adjust, such as individual-level socioeconomic status (SES), which others have proxied by using insurance status. If individual-level SES acts as a confounder, not controlling for it may have biased the association estimated between racial isolation and hypertension. To explore this possibility, we used insurance status (private vs nonprivate) as a proxy for individual-level SES, then restricted the analysis to patients who were not missing information on insurance status, and fit race-stratified models with and without insurance as a covariate (revised n = 49,113 for non-Hispanic black patients and n = 52,556 for non-Hispanic white patients). Lastly, we compared cross-sectional associations for racial isolation (odds ratios and 95% credible intervals) from the model selected based on DIC with the remaining 3 models to investigate whether inference was sensitive to model assumptions.

All statistical analyses were performed by using R version 3.4.4 (The R Foundation). Models were fit by using integrated nested Laplace approximation (25).

## Results

**Figure 1:**
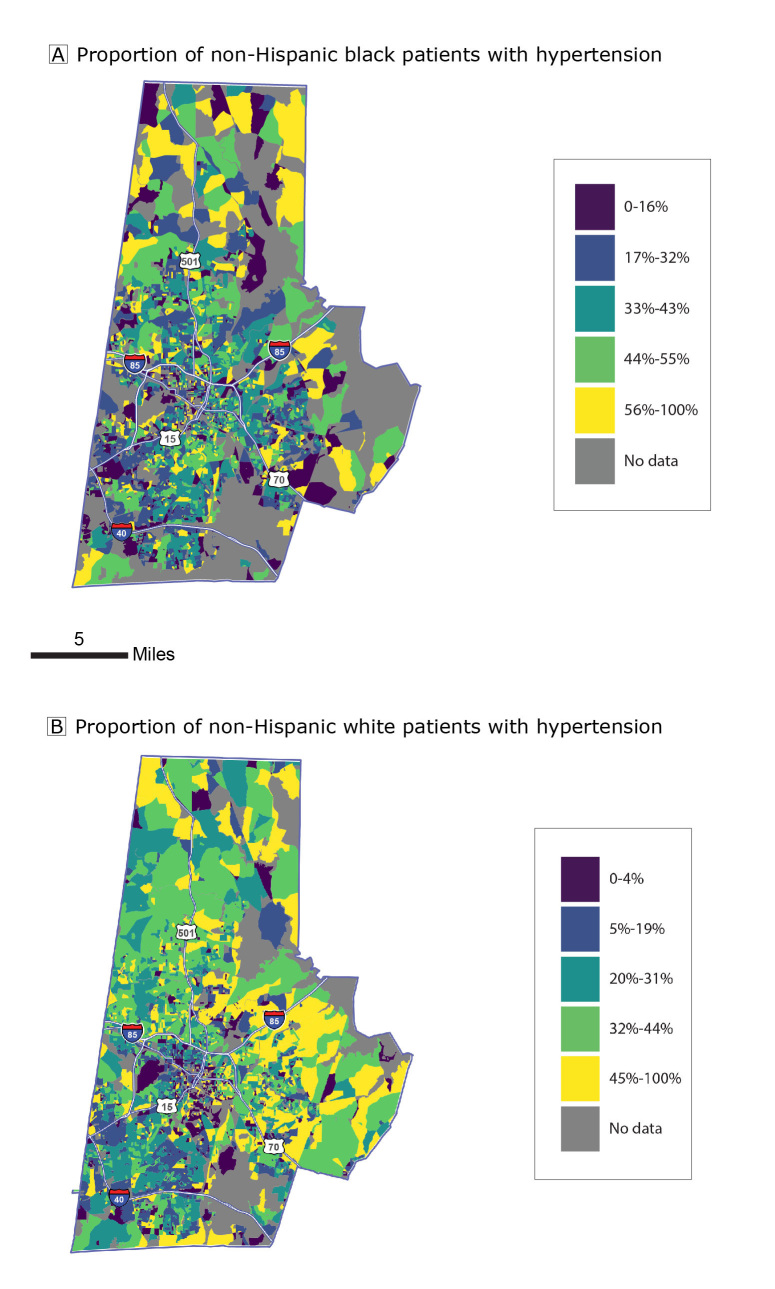
Proportion of patients with hypertension in 2010 Census blocks, by quintile, Duke Medicine Enterprise Data Warehouse patient data, Durham, North Carolina. A, Non-Hispanic black patients. B, Non-Hispanic white patients.

**Figure 2:**
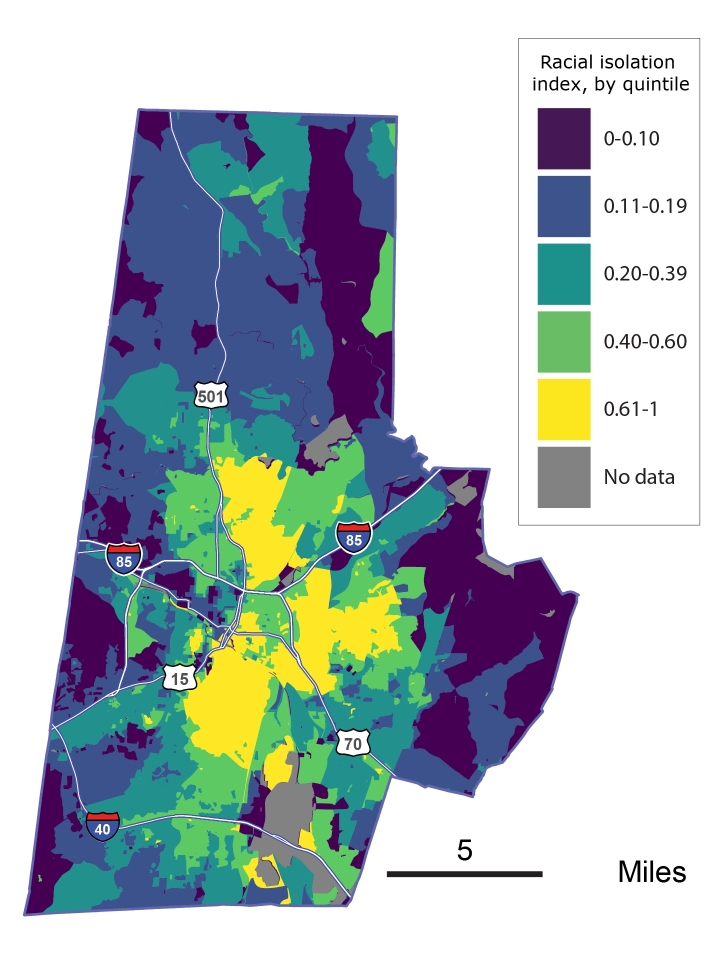
Index value, by quintile, for census-block–level racial isolation of non-Hispanic black residents, Durham, North Carolina. Index of racial isolation is scaled from 0 to 1, with 1 indicating complete isolation.

Patients resided in 3,439 (68%) blocks in the study area. More than half (56%) were non-Hispanic white. Approximately 38% of non-Hispanic black patients and 27% of non-Hispanic white patients had hypertension (Table 1 and Figure 1). In the study area, block-level racial isolation ranged from 0 (no isolation) to 1 (completely isolated), with a mean (standard deviation) of 0.35 (0.21) and median of 0.28 (Figure 2). The mean (standard deviation) racial isolation was 0.54 (0.23) among non-Hispanic black patients and 0.24 (0.17) among non-Hispanic white patients. 

**Table 1:** Summary Statistics of Patient Characteristics in the Duke Medicine Enterprise Data Warehouse Electronic Health Records (n = 147,351), Durham, North Carolina, 2007–2011^a^

Characteristic	Non-Hispanic Black, No. (%)	Non-Hispanic White, No. (%)
Total	65,026 (44.1)	82,325 (55.9)
Hypertension	24,517 (37.7)	21,836 (26.5)
Male	26,157 (40.2)	35,183 (42.7)
**Age, y**		
18–21	6,473 (10.0)	4,205 (5.1)
22–29	10,962 (16.9)	14,680 (18.1)
30–39	12,360 (19.0)	15,392 (18.7)
40–49	12,590 (19.4)	12,436 (15.1)
50–64	14,557 (22.4)	19,626 (23.8)
≥65	8,084 (12.4)	15,986 (19.4)
**Racial isolation, percentile^a^**		
<20th	2,424 (3.7)	27,001 (32.8)
20th–39th	5,952 (9.2)	23,566 (28.6)
40th–59th	11,613 (17.9)	17,638 (21.4)
60th–79th	18,871 (29.0)	10,893 (13.2)
≥80th	26,166 (31.8)	3,227 (5.0)

a The racial isolation index ranges from 0 to 1. In the 3,439 blocks with ≥1 patient in the analysis data set, the 20th, 40th, 60th, and 80th percentiles of racial isolation correspond to racial isolation values of 0.11, 0.21, 0.37, and 0.63, respectively. Data on racial isolation determined by 2010 census block of residence.

### Model choice

For both non-Hispanic black and non-Hispanic white patients, DIC analysis indicated that the spatially structured and convolution models were indistinguishable from one another (difference in DIC ≤5) but preferred over the standard and random-intercept models (Table 2). We chose the spatially structured model over the convolution model for ease of interpretation.

**Table 2:** Comparison of Standard, Random Intercept, Spatially Structured, and Convolution Race-Stratified Logistic Regression Models, Study of Racial Isolation and Spatial Patterning of Hypertension in Durham, North Carolina, 2007–2011^a^

Race	Deviance Information Criterion^b^			
	Standard Model	Random Intercept	Spatially Structured Model	Convolution Model
Non-Hispanic black	63,419	63,327	63,279^c^	63,276^d^
Non-Hispanic white	69,255	68,714	68,417^c^^,^ d	68,417^d^

a All models were adjusted for individual-level patient age and sex and block-level racial isolation of non-Hispanic black patients. Patient data obtained from electronic health records in the Duke Medicine Enterprise Data Warehouse for 2007–2011. Data on racial isolation determined by 2010 census block of residence.

b The deviance information criterion is a generalization of the Akaike information criterion. Taking into account both model fit and model complexity, smaller values indicate a preferred model (22). Using the Watanabe–Akaike information criterion produced the same preferred models.

c The selected model.

d Model with the lowest deviance information criterion value across row.

### Non-Hispanic black patients

Among non-Hispanic black patients, a 0.20 increase in racial isolation was associated with 1.06 (95% credible interval, 1.03–1.10) higher odds of hypertension in the spatially structured model after adjusting for patient age and sex. In the null model, the residual geographic heterogeneity (residual variation on the binomial scale associated with the spatially structured random effect) was approximately 0.36. With the addition of patient age and sex, heterogeneity decreased by 83%, to 0.06. Inclusion of racial isolation further decreased heterogeneity by 33%, to 0.04.

Overall, block-level odds of hypertension among non-Hispanic black patients ranged from 0.62 to 1.88 (Appendix). The blocks with the greatest contributions to hypertension from racial isolation corresponded to areas with higher racial isolation values in central and south central Durham. The magnitude of the association with racial isolation and the width of 95% credible intervals was similar across models (Table 3), although credible intervals in spatial models were wider than those in the aspatial (standard and random intercept) models.

**Table 3:** Odds Ratios (95% Credible Interval) for Hypertension per 0.20-Unit Increase in Racial Isolation, in Race-Stratified Logistic Regression Models, Study of Racial Isolation and Spatial Patterning of Hypertension in Durham, North Carolina, 2007–2011^a^

Race	Standard Model	Random Intercept Model	Spatially Structured Model	Convolution Model
Non-Hispanic black	1.09 (1.07–1.11)	1.08 (1.06–1.10)	1.06 (1.03–1.10)	1.07 (1.03–1.10)
Non-Hispanic white	1.19 (1.17–1.22)	1.19 (1.17–1.23)	1.11 (1.07–1.16)	1.11 (1.07–1.16)

a The standard deviation of racial isolation was 0.17 for non-Hispanic white patients and 0.23 for non-Hispanic black patients. For purposes of comparison, odds ratios are presented per 0.20 racial isolation units. Patient data obtained from electronic health records in the Duke Medicine Enterprise Data Warehouse for 2007–2011. Data on racial isolation determined by 2010 US census block of residence.

### Non-Hispanic white patients

Among non-Hispanic white patients, a 0.20 increase in racial isolation of non-Hispanic black patients was associated with 1.11 (95% credible interval, 1.07–1.16) higher odds of hypertension, after adjusting for patient age and sex. Residual geographic heterogeneity in the null model for non-Hispanic white patients was approximately 0.59. The addition of patient age and sex to the model decreased residual heterogeneity by 66%, to 0.20; the subsequent addition of racial isolation decreased residual heterogeneity by 20%, to 0.16.

Overall, block-level odds of hypertension among non-Hispanic white patients ranged from 0.32 to 2.41 (Appendix). The magnitude of the association with racial isolation was larger in the standard and random-intercept models than in the spatially structured and convolution models (Table 3). The 95% credible intervals were also wider in the spatially structured and convolution models than in the aspatial models.

### Sensitivity analysis

Using the Watanabe–Akaike information criterion instead of DIC would not have resulted in selection of different models (Appendix). The cross-sectional associations between hypertension and block-level proportion of non-Hispanic black residents was smaller than, but not significantly different from, the cross-sectional associations between hypertension and racial isolation. In race-stratified models with and without data on health insurance status, 95% credible intervals for cross-sectional associations for non-Hispanic black and non-Hispanic white patients in the spatially structured model overlapped with those reported in the main analysis (Appendix).

## Discussion

An underlying spatially patterned phenomenon fully characterized residual geographic heterogeneity in hypertension among both non-Hispanic black and non-Hispanic white patients in Durham, North Carolina. Block-level odds of hypertension were more varied among non-Hispanic white patients than non-Hispanic black patients. Patient age and sex accounted for a larger proportion of residual heterogeneity among non-Hispanic black patients than non-Hispanic white patients, whereas the inclusion of racial isolation more similarly proportionately reduced residual geographic heterogeneity among both non-Hispanic black and non-Hispanic white patients. The cross sectional association estimated between racial isolation and hypertension for non-Hispanic white patients was larger than that estimated for non-Hispanic black patients. Furthermore, for non-Hispanic white patients, the cross-sectional associations from aspatial models were larger than those from spatial models. Aspatial models also produced narrower credible intervals than did spatial models.

To date, spatial methods have not been used to study associations between racial isolation and hypertension. We found that non-Hispanic black and non-Hispanic white patients in Durham have, on average, distinct residential contexts, which may lead to separate neighborhood risk factors for hypertension. The exclusive role of the spatially structured random effect in unobserved geographic heterogeneity suggests the presence of local environmental risk factors whose effects on hypertension spill over census-block boundaries.

The larger range in overall block-level odds of hypertension among non-Hispanic white patients than non-Hispanic black patients may indicate underlying differences in race-specific study samples. Non-Hispanic white residents are more spread out than non-Hispanic black residents across Durham, creating more widely varying neighborhood environments for non-Hispanic white residents.

The racial isolation index used in our study measured the geographic separation of black people from other racial/ethnic groups. Non-Hispanic white patients residing in blocks with high values of racial isolation lived in predominantly black neighborhoods and may have greater exposure to neighborhood conditions associated with higher rates of hypertension (eg, unhealthy food environments, poor access to health care). In contrast, when non-Hispanic white patients lived in blocks with low values for racial isolation (which means predominantly white neighborhoods given our definition of racial isolation), they may benefit from health-promoting neighborhood conditions. Non-Hispanic white people who are subject to the same census-block conditions (ie, blocks with high levels of racial isolation) as non-Hispanic black people may be worse off than other non-Hispanic white people because they do not reap neighborhood benefits that provide a health advantage to most other non-Hispanic white people, a premise supported by the findings of others (14). However, the contribution of the spatially structured random effect to overall odds of hypertension suggests that we may not have accounted for other spatially patterned characteristics (eg, healthy food availability) (26).

Our study has several limitations. One is the cross-sectional study design, which precludes causal inference. Second, although we used ICD-9 codes to identify patients with hypertension, we may not have captured data on all patients with hypertension in our study sample. Third, the association observed between racial isolation and hypertension may have been subject to confounding from factors for which we did not control. In the sensitivity analysis, we used insurance status as a proxy for individual-level SES. We observed that inclusion of insurance status, which was missing for approximately 31% of the sample, did not result in significantly different estimated associations between racial isolation and hypertension. Another limitation relates to the study sample’s representativeness of Durham County’s population and the generalizability of results. During the study period, approximately 84% of Durham County residents received care from a Duke Medicine provider at least once, but the study sample excluded patients with residential addresses that could not be found or matched in a reference address data set. The nongeocodable patients, who were removed from analysis, may systematically differ from geocodable patients, who were included in the analysis, in characteristics affecting exposure or health or both.

Despite these limitations, our study enriches the existing body of research on links between racial residential segregation and health, specifically hypertension. Researchers have observed associations between racial residential segregation and health (18,27), but only a few studies have examined segregation and hypertension. Of those that have, most were cross-sectional studies that relied on metropolitan-level measures of segregation or used exclusively aspatial models. For spatially dependent health outcomes, a spatial modeling approach yields more conservative inference; significance in the spatial model, with potentially inflated variances, should also imply significance in the nonspatial model (16,28,29). Furthermore, the local spatial measure of block-level racial isolation may be more closely linked than segregation measures estimated at the metropolitan or city level to individual health outcomes because it is a proxy for the concentration of multiple disadvantages into a single, local ecologic space (6).

Spatial analysis provides an innovative mechanism for evaluating the extent to which residual geographic patterning persists after adjusting for variables that may relate to hypertension, some of which may cluster spatially. Here, we identified blocks and areas of Durham County in which spatially correlated latent risk factors other than racial isolation may be associated with hypertension. In blocks with other neighborhood-based spatially patterned risk factors that contribute to hypertension, additional research is needed to identify what these additional neighborhood characteristics are and how they might be addressed to reduce hypertension. We also identified blocks in Durham with the greatest overall odds of hypertension, which can be used to inform targeted interventions to reduce hypertension risk or manage chronic hypertension.

## Appendix Supplemental Information

Details on the calculation of the racial isolation index are provided in the Appendix, which is available at https://rice.box.com/v/BravoetalSupplementalMaterial. Also included in the Appendix are sensitivity analysis results, including model selection results using the Watanabe–Akaike information criterion, cross-sectional associations estimated between racial isolation and hypertension for individuals after controlling for patient-level insurance status in the selected model. The map decompositions show the overall odds of hypertension in addition to the contribution of racial isolation and the spatially structured block-level random effect to overall odds of hypertension for the average non-Hispanic black patient and the average non-Hispanic white patient in each block.
